# The Recovery of Cognitive and Affective Deficiencies Linked with Chronic Osteoarthritis Pain and Implicated Pathways by Slow-Releasing Hydrogen Sulfide Treatment

**DOI:** 10.3390/antiox10101632

**Published:** 2021-10-16

**Authors:** Gerard Batallé, Xue Bai, Enric Pouso-Vázquez, Gerad Roch, Laura Rodríguez, Olga Pol

**Affiliations:** 1 Grup de Neurofarmacologia Molecular, Institut d’Investigació Biomèdica Sant Pau, Hospital de la Santa Creu i Sant Pau, 08041 Barcelona, Spain; gerard.batalle@e-campus.uab.cat (G.B.); xue.bai@e-campus.uab.cat (X.B.); enrique.pousovazquez@e-campus.uab.cat (E.P.-V.); gerard.roch@e-campus.uab.cat (G.R.); laura.rodriguezpe@e-campus.uab.cat (L.R.); 2 Grup de Neurofarmacologia Molecular, Institut de Neurociències, Universitat Autònoma de Barcelona, 08193 Barcelona, Spain

**Keywords:** anxiety, apoptosis, cognitive deficits, depression, grip strength impairments, hydrogen sulfide, osteoarthritic pain, oxidative stress

## Abstract

Chronic osteoarthritis pain is accompanied by several comorbidities whose treatment has not been completely resolved. The anti-inflammatory, analgesic, and antidepressant effects of slow-releasing hydrogen sulfide (H_2_S) donors during osteoarthritic pain have been shown, but their actions in the accompanying memory impairment and anxious-like behaviors have not yet been demonstrated. Using female mice with chronic osteoarthritic pain, the effects of natural, diallyl disulfide (DADS) or synthetic, morpholin-4-ium 4-methoxyphenyl(morpholino) phosphinodithioate dichloromethane complex (GYY4137) slow-releasing H_2_S donors, on associated cognitive and grip strength deficits and anxiodepressive-like behaviors, were assessed. Their effects on specific brain areas implicated in the modulation of pain and emotional responses were also determined. Results demonstrated an improvement in memory and grip strength deficits, as well as in the anxious-like behaviors associated with chronic pain in GYY4137 and/or DADS treated mice. The painkiller and antidepressant properties of both treatments were also established. Treatment with DADS and/or GYY4137 inhibited: oxidative stress in the amygdala; phosphoinositide 3-kinase overexpression in the amygdala, periaqueductal gray matter, and anterior cingulate cortex; protein kinase B activation in the amygdala and infralimbic cortex; up-regulation of inducible nitric oxide synthase in the amygdala, periaqueductal gray matter and infralimbic cortex and apoptotic responses in the amygdala. These results might explain the recovery of memory and grip strength and the inhibition of allodynia and associated anxiodepressive-like behaviors by these treatments. In conclusion, this study revealed new properties of slow-releasing H_2_S donors in cognitive impairment and affective disorders linked with chronic osteoarthritis pain and their effects on the central nervous system.

## 1. Introduction

Osteoarthritis is a degenerative disorder and one of the most common forms of arthritis. It is also one of the most frequent sources of chronic pain, affecting more than 100 million people worldwide. However, despite its high incidence in society, especially in women (35%), its etiology and treatment have not been fully solved [1].

Osteoarthritis is characterized by the progressive loss of articular cartilage, bone, and synovial remodeling, thus leading to subchondral bone alterations and inflammation, and resulting in severe pain and loss of joint function [2,3]. In addition, and as a consequence of the pain and suffering, patients also experience related cognitive and affective disorders, including memory dysfunction and depressive-like and anxiety-like behaviors [4]. These emotional comorbidities further potentiate hypersensitivity and a more rapid cartilage degradation and disability, thus contributing to a worse quality of life in patients [5]. Different studies have suggested that the underlying mechanisms involved in chronic pain processes are also implicated in memory deficits, as seen in patients with fibromyalgia or severe pain who have exhibited relevant alterations in working memory [6,7,8]. Moreover, osteoarthritis and several emotional and cognitive deficits share a common pathophysiology that involves the activation of the phosphatidylinositol 3 kinase (PI3K)-protein kinase B (AKT) pathway, oxidative stress, and increased proinflammatory responses [9,10,11,12]. The PI3K/p-AKT axis is involved in several cellular processes essential to maintaining homeostasis, including cell growth, cell survival, inflammation, and metabolism [13,14]. This pathway is also implicated in the progression of osteoarthritis pain, and its inhibition has been found to attenuate joint damage by restoring cartilage homeostasis, enhancing autophagy, and suppressing inflammation [10]. The PI3K/p-AKT pathway is also associated with negative mood disorders and may be an important target in the neuroprotection for depressive and anxiety syndromes [15,16]. Furthermore, the inactivation of this pathway has been found to additionally improve the cognitive alterations associated with chronic pain [17].

Several studies revealed that oxidative stress takes part in the pathogenesis of osteoarthritis [18,19]. Therefore, oxidative stress as a result of high reactive oxygen species synthesis, modest antioxidant defenses and lipid-rich formation is a negative factor for certain neuropsychological illnesses, such as alterations in learning and memory functions [11], anxiety [20], depression [21], and nociception [22]. Indeed, oxidative stress blockage can prevent the appearance of these behavioral syndromes, and the emotional and cognitive impairments have been manifested under low levels of antioxidant defenses, thus supporting the relationship between oxidative stress and emotional/memory deficits [11,23,24].

It has been demonstrated that in the progress of osteoarthritis, affected chondrocytes and synovial cells overproduce inflammatory mediators such as interleukin-1β and nitric oxide, which accelerate the degradation of cartilage [25]. Nitric oxide synthetized by the inducible nitric oxide synthase (NOS2) is one of the major proinflammatory and destructive mediators in osteoarthritis [26]. Indeed, high levels of NOS2 have been found in the synovial membrane of individuals with osteoarthritis [27]. Moreover, experiments performed on animals have supported this idea by showing that NOS2 inhibition could be an attractive way to treat osteoarthritis [26]. Finally, the excess production of reactive oxygen species and nitric oxide has been linked with apoptotic responses during osteoarthritis [19]. Therefore, the up-regulation of 4-HNE, a marker of oxidative damage, ends up causing increased BAX levels, leading to cellular apoptosis [18]. In this study, we evaluated the expression of 4-HNE and BAX as markers of oxidative stress and apoptosis.

Hydrogen sulfite (H_2_S) is an endogenous gaseous neurotransmitter that takes part in several physiological processes in the central and peripheral nervous systems [28,29]. Accordingly, H_2_S releasing compounds, especially slow-releasing compounds, have been explored as therapeutic agents in several diseases [30,31]. Numerous findings have demonstrated the beneficial anti-inflammatory and protective actions induced by the exogenous administration of H_2_S in the cartilage and chondrocytes in cell cultures [32,33]. Other works have also proven the positive effects of the intra-articular administration of H_2_S donors on rheumatic disease progression [34], cartilage destruction, and oxidative damage in rats [35]. The antinociceptive and antidepressant effects of the systemic administration of H_2_S donors, such as isothiocyanates, in mice with knee osteoarthritis have also been shown [2,36]. Nevertheless, these compounds have failed to inhibit the anxiolytic-like behaviors concomitant with osteoarthritic or neuropathic pain [24,36].

Our objective is to find new treatments that can palliate the memory and grip strength impairments and the anxiodepressive-like behaviors accompanying chronic osteoarthritic pain, and to establish the principal signaling pathways implicated in these actions in specific areas of the central nervous system. Thus, we evaluated the effects of the systemic administration of two slow-releasing H_2_S donors, a natural garlic bioactive component (diallyl disulfide, DADS) and a synthetic compound (morpholin-4-ium 4-methoxyphenyl (morpholino) phosphinodithioate, GYY4137), on nociceptive, grip strength, and memory deficits, as well as on the anxiodepressive symptoms provoked by the intra-articular administration of monosodium acetate (MIA). To examine the probable pathways involved in these actions, we analyzed their effects on the amygdala, periaqueductal gray matter, infralimbic cortex, and anterior cingulate cortex, all of which are highly involved in the modulation of nociception, memory, and emotional disorders [37,38,39,40].

## 2. Materials and Methods

### 2.1. Animals

Experiments were performed with female C57BL/6 mice (21–26 g; 5–6 weeks old), acquired from Envigo Laboratories (Barcelona, Spain). Animals were accommodated under standard light/dark (12/12 h), temperature (22 °C), and humidity (66%) requirements, with free access to food and water. Experiments were done following 7 days of acclimatization to the environmental conditions, performed between 9:00 a.m. and 5:00 p.m., and in compliance with the guidelines of the European Commission’s directive (2010/63/EC) and the Spanish Law (RD 53/2013) regulating animal research, and were approved by the local Committee of Animal Use and Care of the Autonomous University of Barcelona (ethical code: 9863). Every effort was made to reduce the amount and suffering of the animals used.

### 2.2. Induction of Osteoarthritis Pain

Osteoarthritis pain was induced under isoflurane anesthesia conditions (3% induction, 2.5% maintenance) by the intra-articular injection of MIA (Sigma-Aldrich, St. Louis, MO, USA). The right knee joint was shaved and flexed at a 90° angle, and 10 μL of MIA (15 mg/mL) dissolved in saline solution (NaCl 0.9%; SS) was injected. Control animals were injected with the same volume of SS.

### 2.3. Mechanical Allodynia

Mechanical allodynia was evaluated by measuring the hind paw withdrawal response after stimulation with von Frey filaments of different bending forces (0.008–3.5 g). The animals were placed in Plexiglas tubes (20 cm high × 9 cm diameter) with a wire grid bottom, through which the filaments (North Coast Medical, Inc., San Jose, CA, USA) were applied by using the up–down paradigm [41]. The filament of 0.4 g was applied first, and the filament of 3.0 g was used as a cut-off. The strength of the next filament was increased or decreased depending on the animal’s response. The threshold of the response was calculated using an Excel program (Microsoft Iberia SRL, Barcelona, Spain), which includes curve fitting of the data. The animals were habituated to the environment for 1 h before the experiment. Both ipsilateral and contralateral paws were tested.

### 2.4. Measurement of Grip Strength

We used a computerized grip strength meter (Model 47200, Ugo Basile, Varese, Italy) to measure grip strength according to the method reported by [42]. To measure grip strength in the hind paws, the experimenter held the animal by the base of the tail, allowing the mice to grasp the metal bar of the grip strength meter with both hind paws. The metal bar was connected to a force transducer that automatically recorded the peak force of each measurement in grams. For each mouse, the grip strength of the hind limbs was measured in triplicate. To prevent the mice from gripping the metal bar with their forepaws during the test, the animals were first allowed to grasp a wire mesh cylinder with their forepaws. Baseline grip strength values were recorded for each mouse as the average of three determinations before the administration of MIA or SS. This value was considered 100% of grip strength and was employed as a reference for the following determinations.

### 2.5. Cognitive Behavior

Object recognition memory was evaluated in a gray box (44 × 44 cm) with a non-reflecting base and four walls. This task consisted of 4 sessions of 10 min each (habituation, training, and test). On days 1 and 2, the mice were habituated to the empty box. On the 3rd day, for the training session, the mice were placed again in the box and 2 identical objects were presented. Twenty-four hours later, the mice were placed once again in the box, and one of the familiar objects was replaced by a novel object. The time exploring each of the 2 objects (novel and familiar) was filmed. The discrimination index ((time exploring the novel object–time exploring the familiar)/(time exploring novel + familiar) ∗ 100) was utilized as a measure of cognitive behavior, according to [43]. High values of discrimination represent good recognition memory. Mice were habituated to the testing room for 1 h before starting the evaluation, and the equipment was carefully cleaned between subjects.

### 2.6. Depressive Behavioral Tests

The evaluation of the depressive-like behaviors was performed by using the forced swimming test (FST) and tail suspension test (TST), in which the duration of immobility of the animals was quantified according to the methods described by [44,45], respectively.

In the FST, each mouse was placed in a transparent Plexiglas cylinder (25 cm × 10 cm) containing water to a depth of 10 cm at 24 °C ± 0.1 °C. Each animal was subjected to forced swimming for 6 min, and the total duration of immobility was measured during the last 4 min, when the mice showed a sufficiently stable level of immobility.

In the TST, the mice, isolated acoustically and visually, were suspended by the tail from a horizontal wooden bar (35 cm above the floor) using adhesive tape (1 cm from the tip of the tail). The immobility time in seconds was recorded for 6 min.

In both tests, the mice were considered immobile when they remained completely quiet.

### 2.7. Anxiety Behavioral Tests

The anxiety-like behavior was assessed by using the elevated plus maze (EPM) [46], which consists of an X-shape apparatus with four arms, each 5 cm wide and 35 cm long, with two being open and two being closed, with walls 15 cm high. It was elevated off the ground by 45 cm. The animal was placed in the center of the maze facing the open arms, and its behavior was recorded by a digital camera for 5 min. The number of entries into the open and closed arms and the percentage of time they stayed in the open arms were calculated for each animal. The mice were habituated to the testing room for 1 h before starting the evaluation, and the equipment was carefully cleaned between subjects.

All these experiments were performed by experimenters blinded to the experimental conditions.

### 2.8. Western Blot Analysis

Twenty-nine days after MIA or SS injection, the animals were euthanized by cervical dislocation and the contralateral amygdala, periaqueductal gray matter, infralimbic cortex and anterior cingulate cortex were quickly extracted, frozen, and maintained at −80 °C until usage. In this study, we analyzed the expression of 4-HNE, PI3K, p-Akt, NOS2 and BAX. The sonication of tissues was made in cold lysis buffer RIPA Buffer (R0278; Sigma-Aldrich, MO, USA). After solubilization for 1 h at 4 °C, crude homogenates were sonicated for 10 s and centrifuged at 700× *g* for 20 min at 4 °C. The supernatant (60 μg of total protein) was mixed with 4 × Laemmli loading buffer and loaded onto 4% stacking/12% separating sodium dodecyl sulfate polyacrylamide gels. Proteins were electrophoretically transferred onto a polyvinylidene fluoride membrane for 120 min and blocked with phosphate-buffered saline (PBS; P-5493; Sigma-Aldrich, MO, USA) containing 5% nonfat dry milk, Tris-buffered saline with Tween 20 containing 5% bovine serum albumin (BSA; A-7906; Sigma-Aldrich, MO, USA) or 5% nonfat dry milk, and PBS with Tween 20 containing 5% BSA, for 1 h and 15 min. Then, the membranes were incubated with specific rabbit primary antibodies, the anti 4-HNE (1:200; ab46545, Abcam, Cambridge, UK), PI3K (1:150; ab109006, Abcam, Cambridge, UK), phospho-Akt (1:150; 4060S, Cell Signaling Technology, Danvers, MA, USA), total Akt (1:250; 9272S, Cell Signaling Technology, Danvers, MA, USA), NOS2 (1:200; ab15323, Abcam, Cambridge, UK), BAX (1:250; 14796S, Cell Signaling Technology, Danvers, MA, USA) or β-actin (1:5000, ab8227, Abcam, Cambridge, UK) antibodies overnight at 4 °C. Afterward, blots were incubated for 1 h at room temperature with a horseradish peroxidase-conjugated anti-rabbit secondary antibody (GE Healthcare, Little Chalfont, UK), and proteins were detected with chemiluminescence reagents (ECL kit; GE Healthcare, Little Chalfont, UK). Densitometric analysis was done using the Image-J program (National Institutes of Health, Bethesda, MD, USA).

### 2.9. Experimental Procedures

In the first experiments, we evaluated the effects of repetitive intraperitoneal administration, twice daily, of 200 μmol/kg of DADS and 32 μmol/kg of GYY4137, over 3 and 4 consecutive days, on the cognitive and grip strength losses and mechanical allodynia caused by osteoarthritis (*n* = 6–8 animals for each group). The doses of DADS and GYY4137 were chosen in conformity with another research [47]. The effects of the same treatments on associated depressive- and anxiety-like behaviors were further assessed (*n* = 8 animals for each group). In all experiments, SS plus vehicle-treated mice were used as controls.

MIA-injected animals treated with DADS, GYY4137 or vehicle (0.9% SS) were euthanized by cervical dislocation, and the protein levels of 4-HNE, PI3K, p-Akt, NOS2, and BAX in the amygdala, periaqueductal gray matter, infralimbic cortex and anterior cingulate cortex were evaluated by western blot. In these experiments, SS-injected mice treated with vehicle were used as controls (*n* = 3–4 samples per group).

### 2.10. Drugs

DADS and GYY4137 were purchased from Sigma-Aldrich (St. Louis, MO, USA) and dissolved in SS. Both drugs were intraperitoneally administered 1 h before testing, in a final volume of 10 mL/kg, in accordance with our previous pilot studies and other work [47]. Both drugs were prepared before use, and for each group treated with a drug, the respective control group received the same volume of corresponding vehicle.

### 2.11. Statistical Analyses

Data are expressed as the mean values ± standard error of the mean (SEM). We used the GraphPad software (version 8.0) for the statistical analysis. The effects of DADS and GYY4137 on the allodynia, anxiodepressive-like behaviors, grip strength, and memory deficits associated with osteoarthritis pain were analyzed by using a one-way analysis of variance (ANOVA) followed by the Student–Newman–Keuls test. The effects of both treatments on the expression of several proteins in different brain areas were also analyzed by using a one-way ANOVA and the post hoc Student–Newman–Keuls test. A value of *p* < 0.05 was considered significant.

## 3. Results

### 3.1. Treatment with DADS and/or GYY4137 Inhibits Osteoarthritis-Induced Cognitive Impairment, Hind Limb Grip Strength Deficits and Mechanical Allodynia

The effects of the repetitive intraperitoneal administration, twice daily, of 200 μmol/kg of DADS or 32 μmol/kg of GYY4137, over 3 and 4 consecutive days, on mechanical allodynia and the grip strength and cognitive impairments caused by osteoarthritis were evaluated.

**Figure 1:**
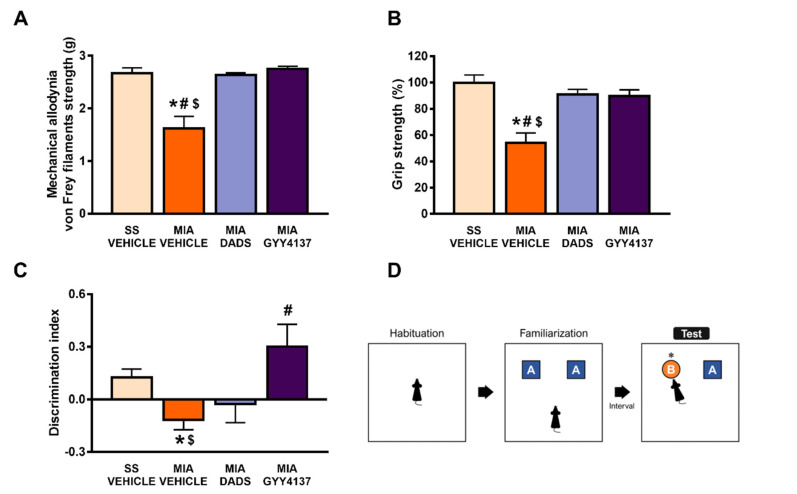
Treatment with DADS and/or GYY4137 inhibited the mechanical allodynia, grip strength, and cognitive deficits induced by monosodium acetate (MIA) injection. The mechanical allodynia, grip strength, and memory deficits induced by osteoarthritis were evaluated on day 29 after MIA or saline solution (SS) injection and treatment with DADS (200 µg/kg, twice daily for 3 days) or GYY4137 (32 µg/kg, twice daily for 4 days) in the von Frey filament strength (g) (**A**), grip strength (%) (**B**) and novel object recognition (discrimination index) (**C**) tests. A schematic representation of the novel object recognition test is shown (**D**). For each test, * denotes significant differences vs. SS-vehicle treated mice, # denotes significant differences vs. MIA-injected mice treated with DADS, and $ denotes significant differences vs. MIA-injected mice treated with GYY4137 (*p* < 0.05; one-way ANOVA followed by the Student–Newman–Keuls test). The results are shown as the mean values ± SEM; *n* = 6–8 animals per experimental group.

Our data demonstrated the complete reversion of the mechanical allodynia (*p* < 0.0001; one-way ANOVA followed by the Student–Newman–Keuls test as compared with SS-vehicle treated mice) (Figure 1A) and grip strength impairments (*p* < 0.0001; one-way ANOVA followed by the Student–Newman–Keuls test as compared with SS-vehicle treated mice) (Figure 1B) provoked by the intra-articular injection of MIA in animals treated with DADS or GYY4137. Both treatments did not have any significant impact on the mechanical allodynia in the contralateral paws of either MIA- or SS-injected mice (data not shown).

Osteoarthritis pain has frequently been associated with memory dysfunction. Our results confirmed these data by showing a decrease in the discrimination index in MIA-injected mice (*p* < 0.0071; one-way ANOVA followed by the Student–Newman–Keuls test vs. SS-vehicle treated mice; Figure 1C). This decrease in the discrimination index was reversed with the GYY4137, but not with the DADS treatment (*p* < 0.001; one-way ANOVA followed by the Student–Newman–Keuls test, as compared with MIA-vehicle treated mice), thus revealing that the impairment of recognition memory associated with chronic osteoarthritis was normalized by the GYY4137 treatment.

### 3.2. The Inhibition of the Anxiodepressive-Like Behaviors of DADS and GYY4137 in Animals with Chronic Osteoarthritis Pain

**Figure 2:**
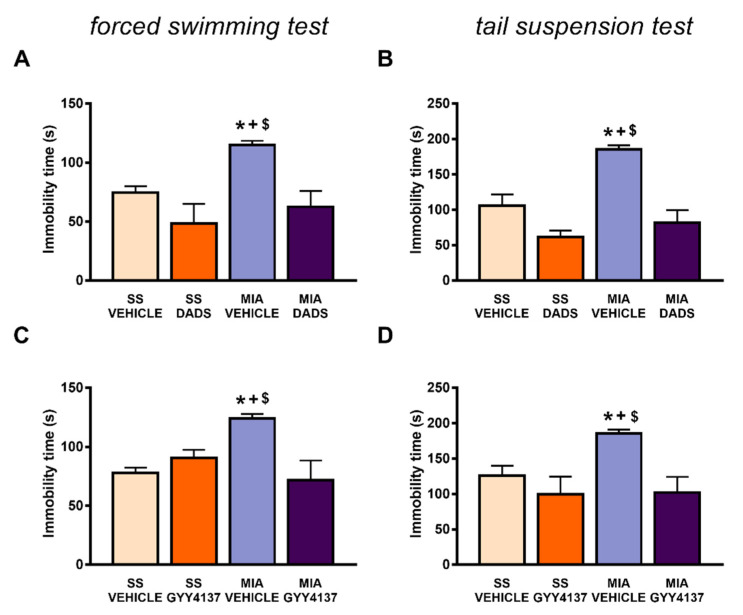
Treatment with DADS and GYY4137 inhibited the depressive-like behaviors linked with chronic osteoarthritis pain. The depressive-like behaviors were evaluated on day 29 after monosodium acetate (MIA) or saline solution (SS) knee injection in mice treated with DADS (200 µg/kg, twice daily for 3 days) or GYY4137 (32 µg/kg, twice daily for 4 days) in the forced swimming test (**A**,**C**) and tail suspension test (**B**,**D**). In the tail suspension and forced swimming tests the immobility time(s) is shown. For each test evaluated, * denotes significant differences vs. SS-injected mice treated with vehicle, + denotes significant differences vs. SS plus DADS or GYY4137, and $ denotes significant differences vs. MIA-injected mice treated with DADS or GYY4137 (*p* < 0.05; one-way ANOVA followed by the Student–Newman–Keuls test). The results are presented as the mean values ± SEM; *n* = 8 animals per experimental group.

We evaluated whether the same administration pattern of both H_2_S slow-releasing donors could normalize the emotive disorders accompanying osteoarthritis pain. At 29 days after MIA injection, depressive-like behaviors were analyzed in the FST and TST, while the anxiety-like behaviors were evaluated in the EPM test. Regarding the depressive-like comportment, both treatments reduced the high immobility time observed in their respective MIA-injected mice treated with vehicle in the FST (*p* < 0.0054; one-way ANOVA followed by the Student–Newman–Keuls test; Figure 2A,C) and TST (*p* < 0.0016; one-way ANOVA followed by the Student–Newman–Keuls test, Figure 2B,D), thus indicating that both H_2_S donors prevented the depressive-like behavior associated with osteoarthritic pain.

**Figure 3:**
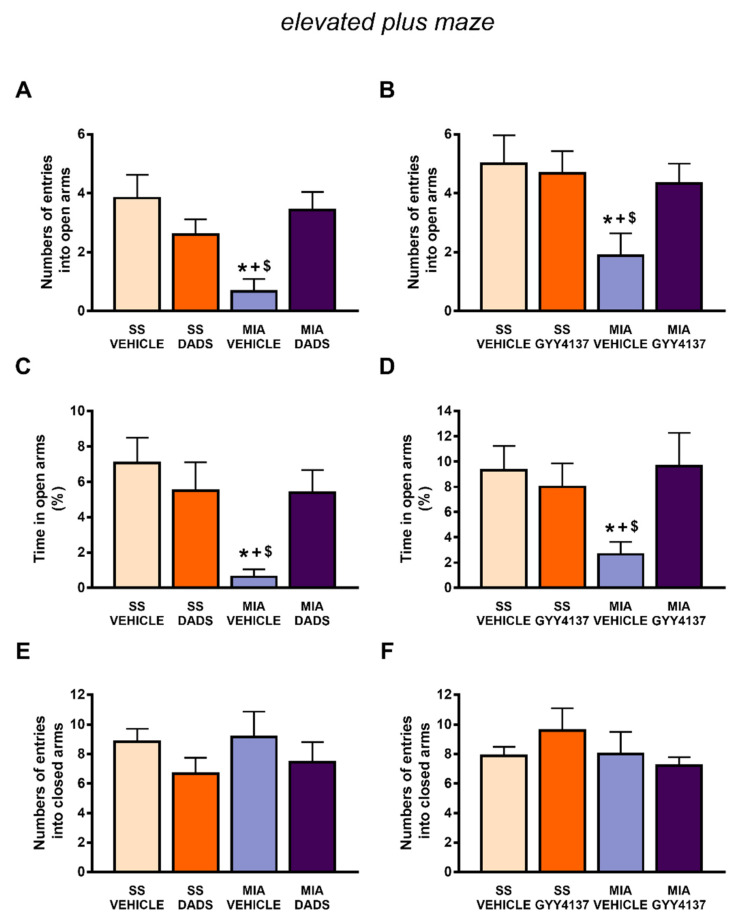
Treatment with DADS and GYY4137 inhibited the anxiety-like behaviors linked with chronic osteoarthritis pain. The anxiety-like behavior was evaluated on day 29 after monosodium acetate (MIA) or saline solution (SS) injection in mice treated with DADS (200 µg/kg, twice daily for 3 days) or GYY4137 (32 µg/kg, twice daily for 4 days) in the elevated plus maze. The number of entries into the open arms (**A**,**B**), percentage of time spent in the open arms (**C**,**D**), and the number of entries into the closed arms (**E**,**F**) are shown. For each response evaluated, * denotes significant differences vs. SS-injected mice treated with vehicle, + denotes significant differences vs. SS plus DADS or GYY4137, and $ denotes significant differences vs. MIA-injected mice treated with DADS or GYY4137 (*p* < 0.05; one-way ANOVA followed by the Student–Newman–Keuls test). The results are presented as the mean values ± SEM; *n* = 8 animals per experimental group.

Osteoarthritis pain is also associated with anxiety-like behaviors, and this was reflected in the significant decrease in the number of entries (*p* < 0.0306 vs. SS-vehicle treated animals, one-way ANOVA followed by the Student–Newman–Keuls test) (Figure 3A,B) and in the time spent in the open arms (*p* < 0.0226 vs. SS-vehicle treated mice; one-way ANOVA followed by the Student–Newman–Keuls test) (Figure 3C,D) of the EPM. These anxiolytic-like responses were normalized through the repetitive administration of DADS (Figure 3A,C) and GYY4137 (Figure 3B,D). All groups of animals exhibited a similar number of entries into the closed arms, suggesting normal locomotor activity in this test independently of the treatments (Figure 3E,F). In summary, both treatments, DADS and GYY4137, inhibited the anxiodepressive-like behaviors accompanying chronic osteoarthritis pain.

### 3.3. Effects of DADS and GYY4137 on the Protein Levels of 4-HNE, PI3K, p-Akt, NOS2 and BAX in the Amygdala of MIA-Injected Mice

**Figure 4:**
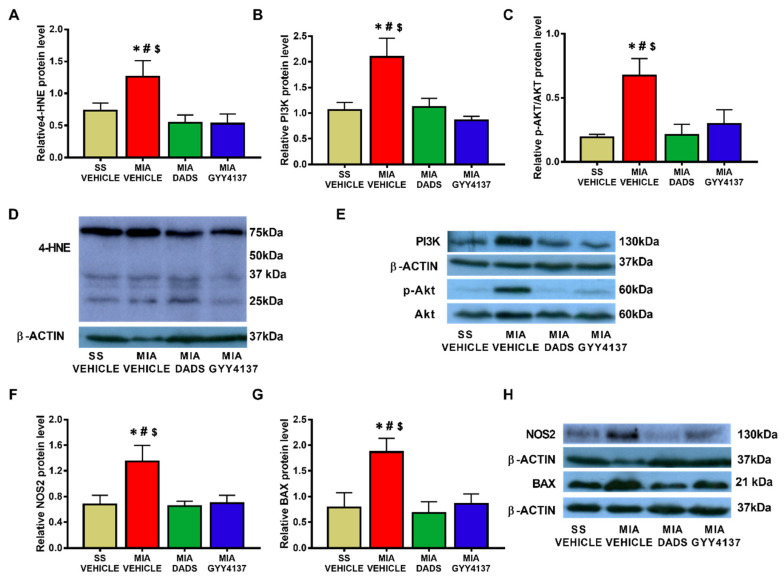
Effects of treatment with DADS and GYY4137 on the expression of 4-HNE, PI3K, p-Akt/Akt, NOS2, and BAX in the amygdala of monosodium acetate (MIA)-injected mice. DADS and GYY4137 treatments normalized the overexpression of 4-HNE (**A**), PI3K (**B**), p-Akt (**C**), NOS2 (**F**), and BAX (**G**) in the amygdala of mice with osteoarthritis pain. Saline solution (SS)-injected mice treated with vehicle were used as controls. Representative blots for 4-HNE (**D**), PI3K and p-Akt/Akt (**E**), NOS2 and BAX (**H**) are shown. All proteins are expressed relative to β-actin levels except P-Akt, which is expressed relative to total Akt. In all graphics, * denotes significant differences vs. SS-vehicle treated mice, # vs. MIA-injected mice treated with DADS, and $ vs. MIA-injected mice treated with GYY4137 (*p* < 0.05; one-way ANOVA followed by the Student–Newman–Keuls test). The results are presented as the mean ± SEM; *n* = 3–4 samples per experimental group.

In the amygdala, our results showed that the intra-articular injection of MIA caused a significant increase in the expression of 4-HNE (*p* < 0.018, one-way ANOVA; Figure 4A), PI3K (*p* < 0.0118, one-way ANOVA; Figure 4B), p-Akt/Akt (*p* < 0.0263, one-way ANOVA; Figure 4C), NOS2 (*p* < 0.0236, one-way ANOVA; Figure 4F), and BAX (*p* < 0.0162, one-way ANOVA; Figure 4G). Treatment with both DADS and GYY4137 normalized the up-regulated protein levels of 4-HNE, PI3K, p-AKT, NOS2, and BAX in this brain area.

### 3.4. Effects of DADS and GYY4137 on the Expression of 4-HNE, PI3K, p-Akt, NOS2 and BAX in the Periaqueductal Gray Matter of MIA-Injected Mice

**Figure 5:**
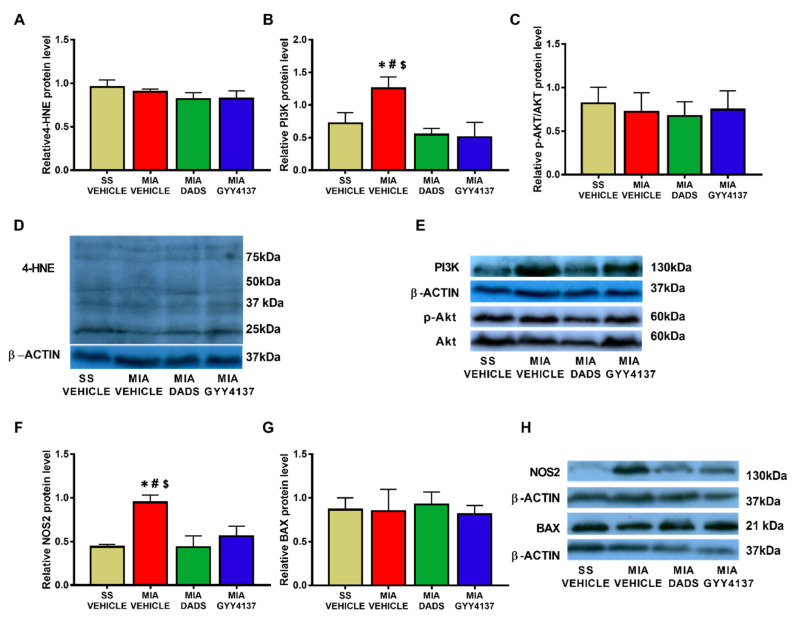
Effects of treatment with DADS and GYY4137 on the expression of 4-HNE, PI3K, p-Akt/Akt, NOS2, and BAX in the periaqueductal gray matter of monosodium acetate (MIA)-injected mice. DADS and GYY4137 treatments normalized the overexpression of PI3K (**B**) and NOS2 (**F**) in the periaqueductal gray matter of mice with osteoarthritis pain. Non-changes in the protein levels of 4-HNE (**A**), p-Akt (**C**), and BAX (**G**) were detected. Saline solution (SS)-injected mice treated with vehicle were used as controls. Representative blots for 4-HNE (**D**), PI3K and p-Akt/Akt (**E**), NOS2 and BAX (**H**) are shown. All proteins are expressed relative to β-actin levels except P-Akt, which is expressed relative to total Akt. In all graphics, * denotes significant differences vs. SS-vehicle treated mice, # vs. MIA-injected mice treated with DADS, and $ vs. MIA-injected mice treated with GYY4137 (*p* < 0.05; one-way ANOVA followed by the Student–Newman–Keuls test). The results are presented as the mean ± SEM; *n* = 3–4 samples per experimental group.

In this brain area, MIA injection provoked a significant increase in the expression of PI3K (*p* < 0.0269, one-way ANOVA; Figure 5B) and NOS2 (*p* < 0.0239, one-way ANOVA; Figure 5F). Treatment with DADS or GYY4137 normalized the enhanced protein levels of PI3K and NOS2 in the periaqueductal gray matter. No changes in the expression of 4-HNE (Figure 5A), p-Akt (Figure 5C), and BAX (Figure 5G) were detected in any of the groups evaluated.

### 3.5. Effects of DADS and GYY4137 on the Protein Levels of 4-HNE, PI3K, p-Akt, NOS2 and BAX in the Infralimbic Cortex of Mice with Osteoarthritic Pain

**Figure 6:**
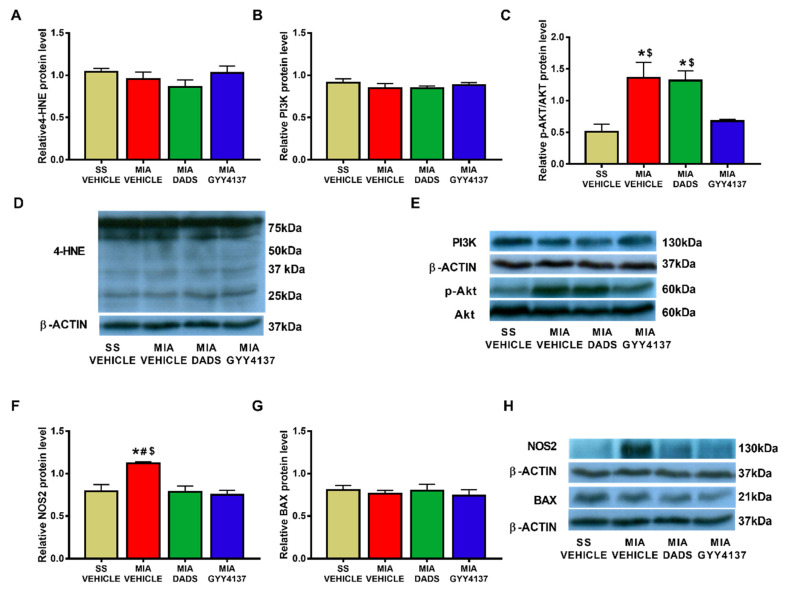
Effects of treatment with DADS and GYY4137 on the expression of 4-HNE, PI3K, p-Akt/Akt, NOS2, and BAX in the infralimbic cortex of monosodium acetate (MIA)-injected mice. DADS and/or GYY4137 treatments normalized the overexpression of p-AKT (**C**) and NOS2 (**F**) in the infralimbic cortex of mice with osteoarthritis pain. The protein levels of 4-HNE (**A**), PI3K (**B**), and BAX (**G**) remained unaltered. Saline solution (SS)-injected mice treated with vehicle were used as controls. Representative blots for 4-HNE (**D**), PI3K and p-Akt/Akt (**E**), NOS2 and BAX (**H**) are shown. All proteins are expressed relative to β-actin levels except P-Akt, which is expressed relative to total Akt. In all graphics, * denotes significant differences vs. SS-vehicle treated mice, # vs. MIA-injected mice treated with DADS, and $ vs. MIA-injected mice treated with GYY4137 (*p* < 0.05; one-way ANOVA followed by the Student–Newman–Keuls test). The results are presented as the mean ± SEM; *n* = 3–4 samples per experimental group.

A significant increase in the expression p-AKT (Figure 6C) and NOS2 (Figure 6F) was observed in the infralimbic cortex of MIA-injected mice (*p* < 0.011, one-way ANOVA; followed by the Student–Newman–Keuls test). While only the GYY4137 treatment normalized the enhanced protein levels of p-Akt, both H_2_S donors prevented the increase in the protein levels of NOS2 in this brain area. No alterations in the expression of 4-HNE (Figure 6A), PI3K (Figure 6B), or BAX (Figure 6G) were observed.

### 3.6. Effects of Treatment with DADS or GYY4137 on the Protein Levels of 4-HNE, PI3K, p-Akt, NOS2 and BAX in the Anterior Cingulate Cortex of MIA-Injected Mice

**Figure 7:**
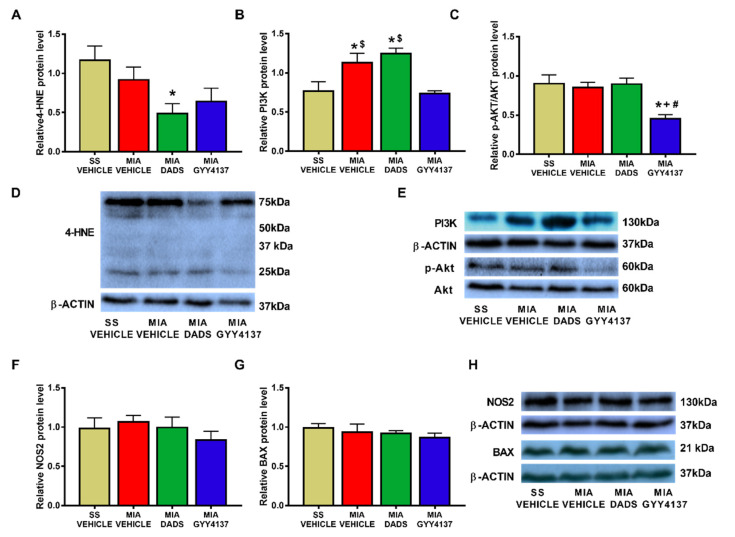
Effects of treatment with DADS and GYY4137 on the expression of 4-HNE, PI3K, p-Akt/Akt, NOS2, and BAX in the anterior cingulate cortex of monosodium acetate (MIA)-injected mice. Treatment with GYY4137 normalized the overexpression of PI3K (**B**). DADS reduced 4-HNE levels (**A**), and GYY4137 diminished the expression of p-Akt (**C**) in the anterior cingulate cortex of mice with osteoarthritis pain. The protein levels of NOS2 (**F**) and BAX (**G**) remained unaltered. Saline solution (SS)-injected mice treated with vehicle were used as controls. Representative blots for 4-HNE (**D**), PI3K and p-Akt/Akt (**E**), NOS2 and BAX (**H**) are shown. All proteins are expressed relative to β-actin levels except P-Akt, which is expressed relative to total Akt. In all graphics, * denotes significant differences vs. SS-vehicle treated mice, + denotes significant differences vs. MIA-injected mice treated with vehicle, # denotes significant differences vs. MIA-injected mice treated with DADS and $ denotes significant differences vs. MIA-injected mice treated with GYY4137 (*p* < 0.05; one-way ANOVA followed by the Student–Newman–Keuls test). The results are presented as the mean ± SEM; *n* = 3–4 samples per experimental group.

Results showed that the proteins levels of PI3K are enhanced in MIA-injected mice (*p* < 0.0033, one-way ANOVA; followed by the Student–Newman–Keuls test), and only GYY4137 can reverse this increase (Figure 7B). Moreover, although MIA did not alter the proteins levels of 4-HNE (Figure 7A) or p-Akt (Figure 7C) in the anterior cingulate cortex, DADS decreased the expression of 4-HNE and GYY4137 down-regulated Akt activation in this brain area. The expression of NOS2 (Figure 7F) and BAX (Figure 7G) remained unaffected in the four groups.

## 4. Discussion

Knee osteoarthritis is a chronic multifactorial disease that causes pain and serious disability problems, as the joints are affected during its development. Currently, there is no specific treatment for osteoarthritis; several pharmacological intervention strategies are available to only alleviate pain symptoms. Moreover, the existing therapies focused on knee regeneration show limited efficacy and, in some cases, significant side effects. Beside this, chronic osteoarthritis pain is known to be also accompanied by different comorbidities, such as alterations in working memory and emotional behaviors; therefore, it is important to palliate all these symptoms in order to achieve a successful treatment.

This study demonstrated the recovery of memory and hind limb grip strength deficits through repetitive treatment with GYY4137 and/or DADS, and further revealed the anxiolytic, antidepressant, and antinociceptive effects induced by both treatments in mice with chronic osteoarthritic pain. These actions seem mainly mediated via inhibiting oxidative stress, PI3K/p-Akt activation, NOS2, and/or BAX over-expression in different brain areas involved in the modulation of nociception and affective disorders, such as the amygdala, periaqueductal gray matter, infralimbic cortex, and/or anterior cingulate cortex.

In accordance with previous studies that have revealed impaired memory function in other chronic pain models [48,49], our study demonstrated a deficit in working memory in MIA-injected animals, as previously shown by [50,51]. Interestingly, the cognitive deficits associated with osteoarthritis pain were completely reduced by the repetitive treatment with GYY4137, but not with DADS, thus highlighting the protective role of GYY4137 compared to DADS in osteoarthritic pain-associated memory impairment. The different effectiveness of both compounds might be a consequence of their different chemical structures, natural (DADS) vs. synthetic (GYY4137) compounds. These results showed the important role of GYY4137, a potent slow-releasing H_2_S donor, in the recovery of memory deficits accompanying osteoarthritis pain.

The anterior cingulate cortex is a region involved in executive, attention, and decision-making processes [52]. Previous studies have reported a direct association between the presence of chronic pain and linked memory loss in this area [39,53,54]. In our pain model, an increased expression of PI3K was observed in the anterior cingulate cortex, which was only reversed by GYY4137 treatment. In addition, and although no changes in the protein levels of 4-HNE or p-Akt were observed in animals with osteoarthritis pain, treatment with DADS significantly decreased the expression of 4-HNE, while GYY4137 diminished that of p-Akt. Considering that common neuroplastic changes associated with chronic pain and emotional disorders have been proposed as important routes for the onset and reciprocal aggravation of both pathologies [55], the inhibition of PI3K/p-Akt induced by GYY4137 in the anterior cingulate cortex might have been responsible for the improvement in working memory observed in the MIA-injected mice treated with this synthetic slow-releasing H_2_S donor. Although DADS decreased the 4-HNE levels, the lack of an effect of this compound on the expression of PI3K/p-AKT in this brain area might explain the non-effects of this treatment on the memory deficits accompanying osteoarthritic pain.

In this study, we also demonstrated the anxiolytic and antidepressant effects of DADS and GYY4137 in animals with chronic osteoarthritis pain. We thus confirmed the antidepressant effects induced by other slow-releasing compounds, such as allyl isothiocyanate and phenyl isothiocyanate, in animals with chronic osteoarthritic [36] or neuropathic pain [24], as well the anxiolytic and antidepressant actions performed by other H_2_S donors, such as sodium hydrosulfide, in different animal models of anxiety or depression without pain [56,57], but in contrast to the non-anxiolytic properties of isothiocyanates during chronic pain [24,36]. The dissimilar chemical structure of isothiocyanates compared to GYY4137 or DADS and/or the different treatment guidelines used could be the most probable reason for these discrepant results. In this study, both treatments were administered twice daily, while isothiocyanates were only administer once daily [36].

The amygdala is closely correlated with the regulation of emotional disorders such as anxiety and depression [38], as well as memory disturbances [58,59]. Our data showed that the elevated levels of 4-HNE, PI3K, p-Akt, NOS2, and BAX in the amygdala of MIA-injected mice were completely normalized by both the DADS and GYY4137 treatments. These data revealed that the oxidative stress, plasticity changes, and inflammatory and apoptotic alterations provoked by knee osteoarthritis in this area were blocked by both H_2_S donors. Thus, considering that one of the main causes of the pathogenesis of osteoarthritis and its associated comorbidities is generated by oxidative stress, PI3K/p-AKT activation, and pro-inflammatory and pro-apoptotic responses [11,25,27], their inhibition with DADS and GYY4137 might possibly explicate their anxiolytic and antidepressant actions in this pain model. These results correspond with other data showing that most of the therapeutic actions of H_2_S may be carried out, at least in part, by reducing reactive oxygen species expression and PI3K/p-Akt/Bcl-2 pathway activation, thus preventing cell apoptosis [35,60,61]. The fact that both treatments inhibited 4-HNE overexpression and the anxiodepressive-like behaviors concurrent with chronic osteoarthritis pain supports the relationship between these disorders and oxidative stress in the amygdala [20,62].

The anterior cingulate cortex also regulates the emotional disturbances associated with pain, such as the anxiety- and depressive-like behaviors [63,64,65,66]. Consequently, and considering that oxidative stress and nociception are related to the development of emotional disorders [9], the fact that DADS and/or GYY4137 modulate the expression of 4-HNE and PI3K/p-Akt in the anterior cingulate cortex might also contribute to the inhibition of anxiodepressive associated with osteoarthritic pain.

Previous studies have reported that the nociceptive, emotional, and cognitive components of pain are also processed in the medial prefrontal cortex, which includes the infralimbic cortex [40]. In this study, we proved that a MIA injection phosphorylated Akt and increased NOS2 expression in the infralimbic cortex, and that both treatments blocked NOS2 overexpression but only GYY4137 inhibited Akt activation, thus suggesting that in this region of the medial prefrontal cortex, DADS has more anti-inflammatory than anti-nociceptive actions; in contrast, GYY4137 diminished the inflammatory and nociceptive responses, thus explaining the higher effectiveness of GYY4137 compared to DADS in modulating the mechanical allodynia and grip strength deficits triggered by knee MIA injection.

The periaqueductal gray matter is an area related to pain modulation [67]. The high levels of PI3K and NOS2 displayed in the periaqueductal gray matter of MIA-injected mice support the fact that this brain area regulated the nociceptive and inflammatory processes implicated in the progression of osteoarthritis pain. Both treatments normalized their over-expression, establishing a causal relationship between the antiallodynic effects and the recovery of hind limb grip strength in DADS- and GYY4137-treated mice during osteoarthritis. In agreement with our results, previous studies have shown the anti-inflammatory effects induced by the knee injection of GYY4137 in another osteoarthritis pain model [35] and with the recovery of the mechanical allodynia and grip strength deficits produced by other slow-releasing H_2_S donors in MIA and complete Freund’s adjuvant-induced osteoarthritis pain [36,68], as well as in animals with nerve-injury- or chemotherapy-induced neuropathic pain [24,69].

## 5. Conclusions

In summary, our results revealed new properties of slow-releasing H_2_S donors in memory impairment and anxiodepressive disorders linked with chronic osteoarthritis pain, as well as their effects on the central nervous system.

## Data Availability

Data is contained within the article.
